# Chitosan Oligosaccharides Suppress Nuclear Factor-Kappa B Activation and Ameliorate Experimental Autoimmune Uveoretinitis in Mice

**DOI:** 10.3390/ijms21218326

**Published:** 2020-11-06

**Authors:** Sheng-Min Hsu, Chang-Hao Yang, Hsien-Yang Tsai, Chia-Jhen Lin, Yi-Hsuan Fang, Chi-Chang Shieh, Shun-Hua Chen

**Affiliations:** 1 Department of Ophthalmology, National Cheng Kung University Hospital, College of Medicine, National Cheng Kung University, Tainan 704, Taiwan; shengmin@ncku.edu.tw (S.-M.H.); towawa1206@gmail.com (C.-J.L.); 2 Department of Ophthalmology, National Taiwan University Hospital, College of Medicine, National Taiwan University, Taipei 100, Taiwan; chyangoph@ntu.edu.tw; 3 Department of Ophthalmology, Tzu Chi Hospital, Taichung 427, Taiwan; tc1512901@tzuchi.com.tw; 4 Department of Microbiology and Immunology, College of Medicine, National Cheng Kung University, Tainan 701, Taiwan; tw7629@gmail.com; 5 Institute of Clinical Medicine, College of Medicine, National Cheng Kung University, Tainan 704, Taiwan

**Keywords:** chitosan oligosaccharides (COS), experimental autoimmune uveoretinitis (EAU), nuclear factor-kappa B (NF-κB)

## Abstract

We investigated the therapeutic potential and mechanism of chitosan oligosaccharides (COS) for experimental autoimmune uveoretinitis (EAU) in mice. EAU was induced in C57/BL6 mice by injection of human interphotoreceptor retinoid-binding protein (IRBP) peptides. At the same time, a high or low dose (20 or 10 mg/kg) of COS or phosphate-buffered saline (PBS) was given to mice daily after EAU induction. We found that mouse EAU is ameliorated by the high-dose COS treatment when compared with PBS treatment. In the retinas of high-dose COS-treated mice, the nuclear translocation of NF-κB subunit (p65) was suppressed, and the expression of several key EAU inflammatory mediators, IFN-γ, TNF-α, IL-1α, IL-4, IL-5, IL-6, IL-10, IL-17 and MCP-1 was lowered. These results suggest that COS may be a potential treatment for posterior uveitis.

## 1. Introduction

Uveitis is a major cause of ocular morbidity and leads to 5–10% of visual impairment worldwide [1,2]. Uveitis can be categorized according to the primary site of inflammation as anterior, intermediate, posterior or panuveitis [3]. Posterior uveitis is the second most common form of uveitis (15–30% cases of uveitis), preceded only by anterior uveitis [1]. However, posterior uveitis tends to damage the photoreceptor cells and leads to permanent blindness more often than anterior uveitis since it occurs in the choroid and adjacent retina. This severe posterior intraocular inflammatory disease is often unrelated to infection and may be associated with the autoimmune responses to unique retinal proteins [1,4]. Current therapies for noninfectious posterior uveitis include many kinds of immunosuppressive agents, such as corticosteroids, antimetabolites and alkylating agents [5]. Due to the nonspecific nature and the severe side effects of these agents, the results of current treatments for noninfectious uveitis remain unsatisfactory [5]. Therefore, more novel agents are needed to control the inflammatory process in posterior uveitis and prevent permanent blindness.

Experimental autoimmune uveoretinitis (EAU), the disease of eye inflammation induced by active immunization with retinal antigens such as interphotoreceptor retinoid-binding protein (IRBP), is the most often used rodent model for the study of autoimmune posterior uveitis [6]. The typical fundal and histological appearance of EAU resembles that of human posterior uveitis, with vasculitis and inflammatory cells infiltrating the vitreous cavity and causing damage to the neurosensory retina layer [7]. Nuclear factor-kappa B (NF-κB) plays an important role in inflammation induction, and several previous studies have found that NF-κB is activated in EAU [8,9]. Therefore, inhibition of NF-κB activation can ameliorate EAU [8,9]. The levels of several NF-κB regulated inflammatory mediators, such as interleukin (IL)-1, IL-6, tumor necrosis factor (TNF)-α and monocyte chemoattractant protein (MCP)-1, are increased in the EAU animal model and can be modulated by suppression of NF-κB activation [7,10,11,12].

Chitosan oligosaccharides (COS) are a mixture of oligomers of β-1,4-linked D-glucosamine residues with abundant levels in the exoskeletons of crustaceans and cell walls of fungi and insects [13,14]. COS have many well-known biological effects such as antitumor, antibacterial, anti-inflammation, antioxidative and antiapoptotic activities [15,16,17,18]. COS are nontoxic and biodegradable and have been used as a bioactive material [13]. Previous studies have shown that COS can inhibit NF-κB activation, attenuate oxidative stress-related retinal degeneration, experimental autoimmune anterior uveitis (EAAU), and prevent retinal ischemia and reperfusion injury in rats [13,19,20]. COS can penetrate the blood–brain barrier of mice [21]. Therefore, COS should reach the retina since the retina is a part of the central nervous system [22]. However, the effect of COS for the anti-inflammatory activity in a mouse model of EAU remains unknown. In this study, we investigated the therapeutic effect and possible mechanism of COS in EAU mice. Here, we showed the effectiveness of COS in ameliorating IRBP-induced EAU in mice.

## 2. Results

### 2.1. COS Significantly Decreased EAU in Mice

**Figure 1:**
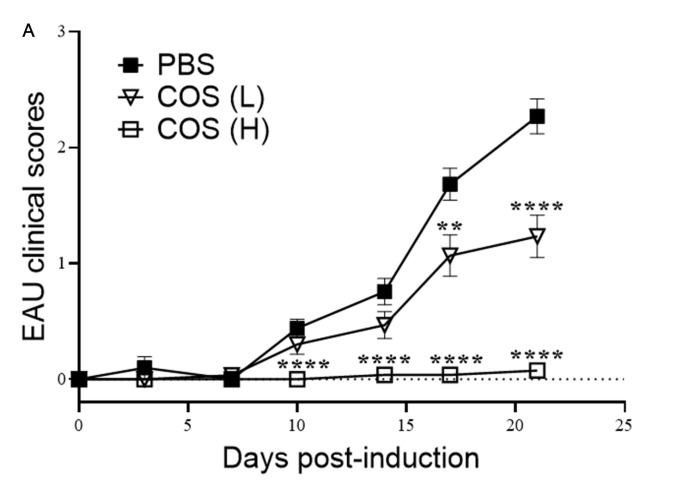

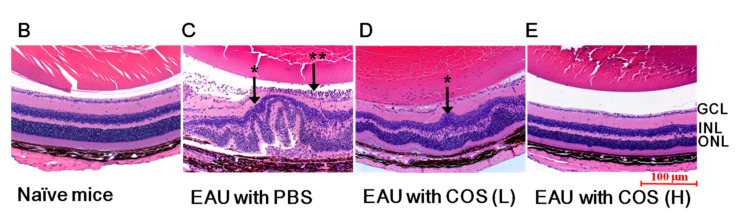
Chitosan oligosaccharides (COS) treatment reduces experimental autoimmune uveoretinitis (EAU) severity in mice. (**A**) Diseases scores of mice with EAU induction and treated with the high-dose (20 mg/kg) of COS (*n* = 27) or low-dose (10 mg/kg) of COS (*n* = 30) or phosphate-buffered saline (PBS) (*n* = 41). Data show the means ± SE values (error bars). ** *p* < 0.01, **** *p* < 0.0001, compared with PBS-treated mice at the same time point. Representative photomicrographs of hematoxylin and eosin (H&E)-stained slides of the retinas of (**B**) naïve C57BL/6 mice without EAU induction, (**C**) EAU mice treated with PBS, (**D**) EAU mice treated with the low-dose (10 mg/kg) of COS and (**E**) EAU mice treated with the high-dose (20 mg/kg) of COS. * indicates retinal folds; ** indicates leukocytes in the vitreous cavity. Images are the representative results of at least four samples per group from two independent experiments. GCL, ganglion cell layer; INL, inner nuclear layer; ONL, outer nuclear layer.

EAU was induced in mice by injecting IRBP_1-20_ emulsified with complete Freund’s adjuvant subcutaneously and pertussis toxin intraperitoneally. The high or low dose (20 or 10 mg/kg) of COS was injected intraperitoneally into the mice daily from days 0–21 post EAU induction. The group of mice which received phosphate-buffered saline (PBS) instead of COS served as controls. In the PBS-treated group, the mouse eye showed signs of inflammation starting 10 days after EAU induction and continued to deteriorate over the following two weeks with a peak 21 days after induction (Figure 1A). Mice that received the high-dose of COS treatment exhibited a significant delay in disease onset and low EAU scores over time. After EAU induction for 21 days, the disease incidences of both PBS- and low-dose COS-treated groups were high (32 of 41 and 13 of 30, respectively) (Table 1). The disease incidence of the high-dose COS-treated group (0 of 27) was significantly lower than that of the PBS-treated group (*p* < 0.005). The mean clinical severity score of PBS-treated mice (2.30 ± 0.16) was significantly higher than those of mice treated with the low- or high-dose of COS (1.20 ± 0.18 and 0.10 ± 0.06, respectively) (*p* < 0.0001). The difference in the mean clinical severity scores of mice treated with the low- or high-dose of COS was also statistically significant (*p* < 0.0001). The fact that the majority of mice given the high-dose of COS had peak scores of 1 or lower (i.e., mild or no disease) indicates the suppressive activity of COS on EAU. In addition, examination of hematoxylin and eosin (H&E)-stained, paraffin-fixed slides revealed that the retinal sections of eyes from EAU mice that received the high-dose COS displayed a decrease of cell infiltration into the vitreous cavity without the retinal folds in retinal layer structures observed in the PBS-treated mice (Figure 1B–E and Supplementary Figure S1). There was no mortality or extraocular morbidity associated with the COS treatment in the experimental mice. There was also no tumor growth or infection after COS treatment in our study.

### 2.2. The Influence of COS Treatment on Inflammatory Mediators in the Retinas of EAU Mice

**Figure 2:**
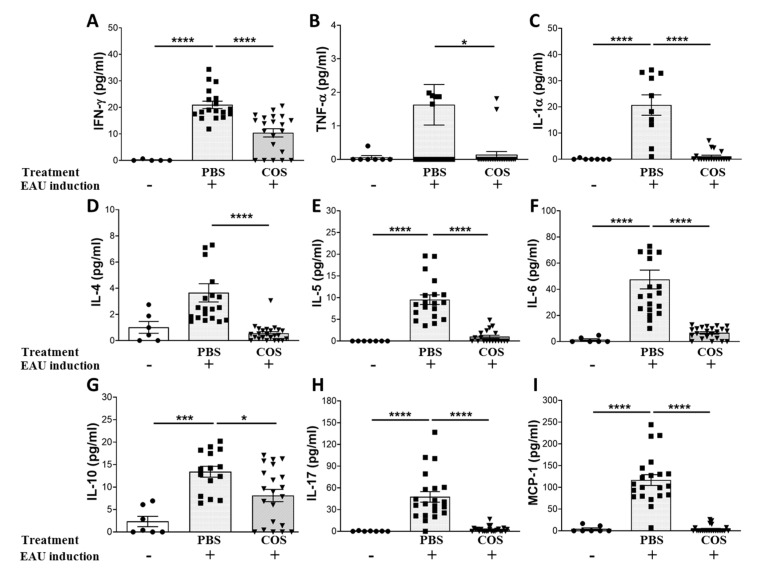
The influence of COS treatment on inflammatory mediators in the mouse retinas. Levels of IFN-γ (**A**), TNF-α (**B**), IL-1α (**C**), IL-4 (**D**), IL-5 (**E**), IL-6 (**F**), IL-10 (**G**), IL-17 (**H**) and monocyte chemoattractant protein (MCP)-1 (**I**) in the retinas of mice with (+) or without (-) EAU induction and treated with PBS or COS (20 mg/kg) are shown. Data show the means ± SE values (error bars) of > 8 samples per group. * *p* < 0.05, *** *p* < 0.001, **** *p* < 0.0001.

We then measured the levels of inflammatory mediators in the retinas of naïve mice and EAU mice with different treatments using the Luminex assay. The levels of IFN-γ, TNF-α, IL-1α, IL-4, IL-5, IL-6, IL-10, IL-17 and MCP-1 in the retinas of PBS-treated EAU mice were much higher than those of naïve mice (Figure 2A–I). Importantly, the levels of these inflammatory mediators in the retinas of EAU mice treated with the high-dose of COS were significantly lower than those treated with PBS (*p* < 0.05 in all paired comparisons).

### 2.3. Decreased Expression of IFN-γ, TNF-α and MCP-1 in the Retinas of COS-Treated EAU Mice

**Figure 3:**
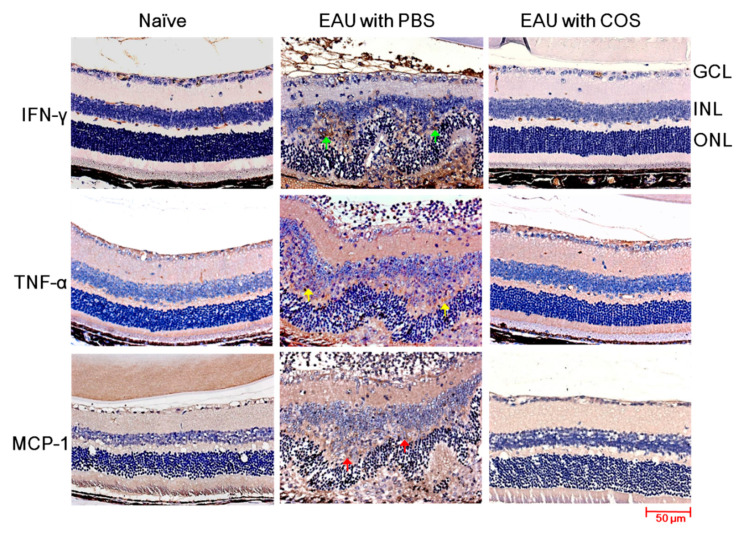
Immunohistochemical examination of inflammatory mediators in the mouse retinas. Eyes of mice without EAU induction and treatment (naïve) or with EAU induction and treated with PBS or COS (20 mg/kg) were examined by staining for the indicated inflammatory mediators. The retinal portion is shown. The green, yellow, and red arrows indicate the cells positive for IFN-γ, TNF-α, and MCP-1, respectively. Images are representative of more than four samples per group from two independent experiments. GCL, ganglion cell layer; INL, inner nuclear layer; ONL, outer nuclear layer.

Based on the results of retinal cytokine expression, we performed immunohistochemical studies on the expression of proinflammatory cytokines and chemokine (IFN-γ, TNF-α and MCP-1) to reveal the changes in tissue inflammation. The retinas of PBS-treated EAU mice showed increased expression of IFN-γ in the inner nuclear layer (INL) and outer nuclear layer (ONL) and infiltrating leukocytes when compared with those of naïve mice (Figure 3, first row). The expression of IFN-γ was reduced markedly in the retinas of EAU mice treated with the high-dose of COS when compared with PBS treatment. The results of the immunohistochemical staining and Luminex analyses were consistent. Staining results of TNF-α and MCP-1 also showed that EAU induction markedly increased the expression of these two proteins in the inner nuclear layer (INL) of retinas and infiltrating leukocytes (Figure 3, lower two rows) and that the high-dose of COS treatment markedly decreased these two proteins in mouse retinas. Therefore, treatment with COS suppressed IFN-γ, TNF-α and MCP-1 in the retinas of mice with EAU induction.

### 2.4. COS Suppresses NF-κB Activation in Retinas of Mice with EAU Induction

**Figure 4:**
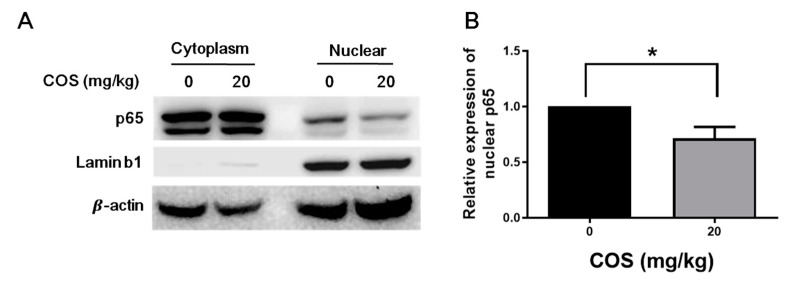
COS inhibits the nuclear translocation of nuclear factor-kappa B (NF-κB) subunit p65 in the retinas of mice with EAU induction. Mice were treated with or without COS (20 mg/kg) for 21 days and assayed for the nuclear and cytoplasmic fractions of NF-κB subunit, p65, β-actin and lamin b1 (nuclear marker) by Western blotting. (**A**) The representative blots and (**B**) quantitated results are shown. The nuclear p65/β-actin value of the retinas without COS treatment was set as 1. Data show the means ± SE values (error bars) of 4 samples per group. * *p* < 0.05.

NF-κB activation is demonstrated by the translocation of its component p65 to the nucleus and can promote the expression of cytokines and chemokines, such as IL-1, IL-6, TNF-α and MCP-1 [23,24]. To test whether COS can reduce NF-κB activation, the nuclear and cytoplasmic proteins in the retinas of mice with EAU induction and treated with or without COS were extracted and analyzed by Western blotting (Figure 4A). The nuclear translocation of p65 was noted after EAU induction. After treatment with the high-dose of COS, the level of nuclear translocation of p65 was reduced significantly (*p* < 0.05) (Figure 4B), suggesting that COS diminishes NF-κB activation in the retinas of mice with EAU induction.

**Figure 5:**
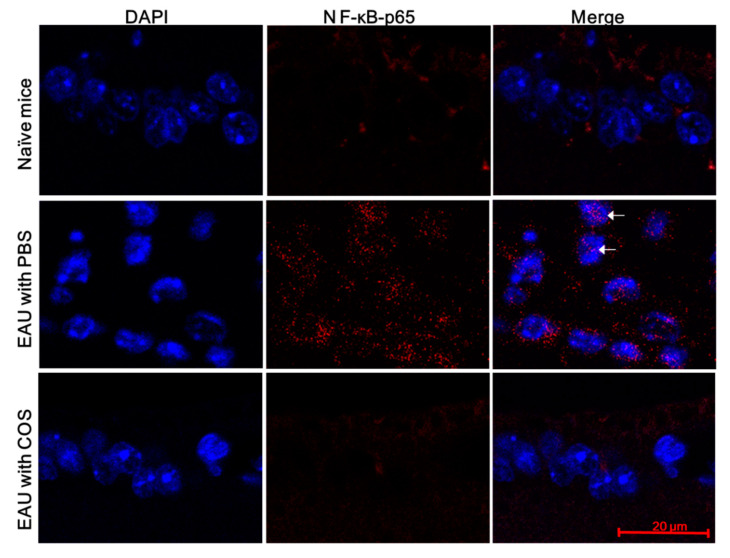
COS treatment reduces the nuclear translocation of NF-κB subunit p65 in the retinas of mice with EAU induction. Eyes of mice without EAU induction and treatment (naïve) or with EAU induction and treated with PBS or COS (20 mg/kg) were monitored by immunofluorescence staining for NF-κB (p65) with antibody and nuclei with DAPI. The ganglion cell layer of the retina is shown. Images are representative of > 4 samples per group from two independent experiments. White arrows indicate NF-κB (p65) in the nucleus.

We also monitored p65 in nuclei using immunofluorescence staining. A low level of p65 was detected in the retinas of naïve mice without EAU induction (Figure 5 and Supplementary Figure S2, upper row). After EAU induction for 21 days, abundant p65 was detected in the ganglion cell layer of the retinas of mice, and p65 was located in the nuclei of some retinal cells (Figure 5 and Supplementary Figure S2, middle row). Treatment with the high dose of COS decreased both p65 expression and nuclear translocation in the retinas of mice with EAU induction (Figure 5 and Supplementary Figure S2, lower row). Results of NF-κB Western blotting and immunofluorescence obtained from mouse retinas indicate that EAU enhanced NF-κB activation, and COS treatment inhibited NF-κB activation.

## 3. Discussion

Our study demonstrated that COS could effectively ameliorate the clinical severity of uveoretinitis and reduce the expression of inflammatory mediators in mice with EAU. We also found that COS reduced NF-κB activation by suppressing p65 subunit translocation to the nucleus, which may contribute to the decreased proinflammatory cytokines and chemokine production in EAU. Our results suggested that COS treatment could exert anti-inflammatory effects in EAU by suppression of NF-κB activation and cause a reduction in the expression of inflammatory mediators, which in turn decreased trafficking and recruitment of inflammatory cells to the vitreous and retina, leading to protection of the retina from damage. Our study showed the therapeutic potential of COS in the treatment of human noninfectious posterior uveitis in the future.

NF-κB is a well-known transcription factor that could regulate the expression of genes involved in ocular inflammation [11,12]. Many studies have shown that NF-κB has a pivotal role in EAU and that inhibition of NF-κB activation can reduce the levels of tissue inflammation by lowering the inflammatory mediators and cell infiltration into the vitreous and retina [11,12]. Several studies have indicated that the expression of cytokines and chemokine, such as TNF-α, IL-1, IL-6 and MCP-1, is governed by NF-κB [23,24]. We previously demonstrated significant activation of NF-κB in the retina during EAU, and the proteasome inhibitor bortezomib effectively reduced ocular inflammation in EAU [11]. In this study, we demonstrated that COS could also suppress NF-κB activation and decrease the expression of IL-1, IL-6, TNF-α and MCP-1, resulting in ameliorated uveoretinitis. Fang et al. have reported that COS exhibits anti-inflammatory activities by suppressing NF-κB activation in vitro and in vivo [13]. They have reported that COS suppressed NF-κB activation in rat models of EAAU [13]. In this study, we demonstrated that COS treatment effectively inhibited NF-κB activation and the production of multiple cytokines and chemokine in the mouse model of EAU. However, the suppressive effects of COS on retinal NF-κB may be an indirect effect since COS was administered systemically and not locally.

Being an autoantigen-induced autoimmune disease, EAU is with inflammation dominated by acute inflammatory mediator responses [7]. In this study, we found that levels of inflammatory mediators, such as IFN-γ, TNF-α, IL-1α, IL-4, IL-5, IL-6, IL-10, IL-17 and MCP-1, were increased significantly in the retinas of PBS-treated EAU mice when compared with those in naïve mice (Figure 2). TNF-α is a major proinflammatory cytokine and plays a central role in autoimmune uveoretinitis [7]. TNF-α antagonists have been used clinically to treat ocular inflammatory disorders successfully [25,26]. In this study, we found that COS treatment in EAU mice could suppress not only TNF-α but also many other inflammatory mediators, such as IFN-γ, IL-1α, IL-4, IL-5, IL-6, IL-10, IL-17 and MCP-1, in retinas. Therefore, we expect that COS, which may reduce multiple inflammatory cytokines and chemokine through suppressing NF-κB activation, appears to be a better anti-inflammatory agent for the treatment of autoimmune uveoretinitis than TNF-α antagonists.

It is well known that overproduction of reactive oxygen species (ROS) results in accumulative oxidative damage to cellular components, alters many cellular functions and leads to apoptotic cell death [27,28]. Excessive ROS produced in the body are scavenged by antioxidant enzymes, which are important defense mechanisms in controlling the levels of ROS and preventing oxidative cell death [29,30]. Fang et al. found that COS treatment in mice increased superoxide dismutase and catalase activities and glutathione levels [19]. This was accompanied by decreases in ROS production and cellular damage. We have previously found that suppression of the reactive oxygen response can alleviate EAU in mice [12]. Actually, COS has been recommended as a superior agent to other antioxidants because of its biocompatibility, biodegradability, nontoxicity and absorption properties [31,32]. In addition, COS is a natural product abundant in marine foods helpful for the prevention and treatment of diseases [19]. Therefore, the antioxidative effect may represent another protective mechanism of COS treatment in EAU.

In this study, the mice were treated with COS from the same day when EAU was induced to ensure that the initiation of COS treatment during the early stage of EAU development. We have not evaluated the drug’s efficacy when it is applied after EAU is full-blown, as usually is the case in clinic settings. At the end of the experiment after COS treatment, there was no mortality, tumor growth or severe infection noted. However, the systemic adverse effects of COS treatment deserve meticulous consideration.

## 4. Materials and Methods

### 4.1. Mice

Eight- to twelve-week-old female C57BL/6J mice obtained from the Laboratory Animal Center in our college were used for experiments. All experiments were performed according to the protocol approved by the Institutional Animal Care and Use Committee of National Cheng Kung University (with the approval number of 105023, 20 October 2015) and to the statement of the Association for Research in Vision and Ophthalmology (ARVO) for the Use of Animals in Ophthalmic and Vision Research.

### 4.2. Induction and Treatment of EAU

EAU was induced as previously described [11,33]. Briefly, mice were immunized with 100 μL of an emulsion of PBS containing 200 μg of human IRBP peptide 1–20 (hIRBP_1-20_) (GPTHLFQPSLVLDMAKVLLD) and complete Freund’s adjuvant containing 500 μg of inactivated *Mycobacterium tuberculosis* H37RA (Difco Laboratories, Detroit, MI, USA). Mice received the emulsion at two sites on the lower back, followed by an intraperitoneal injection of 1.5 μg pertussis toxin as an additional adjuvant. Mice were treated with COS at the doses of 10 or 20 mg/kg in 0.1 mL of PBS by intraperitoneal injection every day starting on the day of EAU induction. The ocular fundus of mouse eyes was examined by slit lamp twice a week for 21 days for clinical signs of EAU. After anesthesia of mice, both mouse pupils were dilated with tropicamide and phenylephrine hydrochloride ophthalmic solutions. The severity of inflammation was clinically graded on a scale of one to five as described previously [11,34]: 0, no inflammation; 1, ≤5 focal vasculitis spots or soft exudates; 2, linear vasculitis or spotted exudates in <50% of the retina; 3, linear vasculitis or spotted exudates in ≥50% of the retina; 4, retinal hemorrhage or severe exudates and vasculitis; 5, exudative retinal detachment or subretinal (or vitreous) hemorrhage. A mouse was considered to have uveoretinitis if at least one of its eyes had a score of two or more. The severity of uveoretinitis is represented as the highest clinical score achieved by either eye in a mouse.

### 4.3. Histopathological Evaluation

Mouse eyes were collected at the peak of the clinical response (21 days after induction of EAU), immersed in 10% formaldehyde and then stored until being processed. Fixed and dehydrated tissues were embedded in paraffin, and 3 μm sections were cut through the cornea–optic nerve plane and then stained with H&E. Presence or absence of disease was evaluated in a blinded fashion by examining six sections cut at different levels for each eye. Leukocytes infiltration into the vitreous cavity and retinal folding were considered as uveoretinitis.

### 4.4. Immunohistochemical and Immunofluorescence Staining of Inflammatory Mediators and p65 in Mouse Retinas

The sections were obtained from the same paraffin blocks used for histological evaluation. After deparaffinization with xylene solutions and rehydration with a graded series of ethanol in PBS, 0.3% hydrogen peroxidase was added to block the intrinsic peroxidase activity. The specimens were then rinsed with 5% normal mouse serum and incubated overnight with polyclonal antibodies against IFN-γ, TNF-α or MCP-1 (Abcam, Cambridge, UK). Then, the biotinylated secondary antibody against rabbit IgG and an avidin-biotinylated peroxidase complex (Abcam) were applied with 3,3′-diaminobenzidine as a peroxidase substrate. The sections were counterstained with hematoxylin, dehydrated, and embedded. Specimens stained without the primary antibody were used as negative controls. Eye sections were also monitored by immunofluorescence staining with the antibody against mouse p65 (Abcam) and DAPI (4′,6-diamidino-2-phenylindole) for DNA.

### 4.5. Luminex Assay

Eyeballs were enucleated from euthanized mice and cut at the equator around the ora serrata. The posterior pole of the eyes was separated from the anterior pole and lens. From the posterior pole, the neurosensory retina was extracted from the retinal pigment epithelial layer. The extract from six mouse retinas was placed in 300 μL of 0.5% NP-40 (Abcam) on ice (one min) and briefly sonicated five times for 10 s at the probe intensity of 7 (MicrosonTM XL2000 Ultrasonic liquid processor, Qsonica, LLC, Newton, CT, USA). After removal of insoluble material by centrifugation (200× *g* for 5 min), the protein concentration of the retinal lysate was measured at 280 nm on an ND-1000 spectrophotometer. The retinal lysate was used for quantification of IFN-γ, TNF-α, IL-1α, IL-4, IL-5, IL-6, IL-10, IL-17 and MCP-1 by the murine multiplexing bead immunoassay (Invitrogen, Carlsbad, CA, USA) according to the instructions of the manufacturer. Briefly, 25 μL of retinal samples in PBS was incubated with antibody-coupled beads. After a series of washes, a biotinylated detection antibody was added to the beads, and the reaction mixture was detected by the addition of streptavidin–phycoerythrin. The bead set was analyzed by a flow-based Luminex 200 suspension array system (Luminex Corporation, Austin, TX, USA).

### 4.6. Statistical Analysis

Data are shown as the mean ± SEM. For statistical comparison, EAU disease scores and Luminex analysis were analyzed with one-way ANOVA followed by Holm–Sidak test. EAU incidences were analyzed by chi-squared test. Quantitation of Western blotting was analyzed by Student’s *t*-test. All analysis was analyzed with Prism 8.0 software. A *p* value of <0.05 was considered statistically significant and * indicates *p* < 0.05, ** indicates *p* < 0.01, *** indicates *p* < 0.001 and **** indicates *p* < 0.0001.

## 5. Conclusions

In summary, we have demonstrated that COS can ameliorate EAU in mice. Reduced intraocular inflammation was associated with decreased expression of many inflammatory mediators. These effects are achieved by inhibiting the activation (nuclear translocation) of NF-κB in the mouse retinas. Our results show the potential of COS as an effective treatment for autoimmune uveoretinitis in the future.

## Figures and Tables

**Table 1:** Effects of COS treatment on EAU ^a^.

Treatment	Incidence of Mice with Clinical Score ≧ 2	Mean Peak Disease Score
PBS	32/41	2.30 ± 0.16
COS (L) ^b^	13/30	1.20 ± 0.18 ^2^
COS (H) ^c^	0/27 ^1^	0.10 ± 0.06 ^3,4^

^a^ Data are compiled from four experiments in which similar results were obtained. ^b^ Low-dose COS treatment (10 mg/kg). ^c^ High-dose COS treatment (20 mg/kg). ^1^, *p* < 0.005, between PBS and COS (H) groups. ^2^, *p* < 0.0001, between PBS and COS (L) groups. ^3^, *p* < 0.0001, between PBS and COS (H) groups. ^4^, *p* < 0.0001, between COS (H) and COS (L) groups.

## Supplementary Materials

The following figures are available online at https://www.mdpi.com/1422-0067/21/21/8326/s1. Figure S1: The lower magnification images of Figure 1B–E. Figure S2: The lower magnification images of Figure 5.

## Abbreviations

COS	Chitosan oligosaccharides
PBS	Phosphate-buffered saline
ROS	Reactive oxygen species
EAU	Experimental autoimmune uveoretinitis
IRBP	Interphotoreceptor retinoid-binding protein
IL	Interleukin
IFN	Interferon
TNF	Tumor necrosis factor
MCP	Monocyte chemoattractant protein
