# Supplementary material for: Chitosan Oligosaccharides Suppress Nuclear Factor-Kappa B Activation and Ameliorate Experimental Autoimmune Uveoretinitis in Mice
Source: Int J Mol Sci. 2020 Nov 6;21(21):8326. doi: 10.3390/ijms21218326 (PMC7664198; doi:10.3390/ijms21218326)
Supplement: Supplementary file 1 [file ijms-21-08326-s001.pdf]

International Journal of
Molecular Sciences

MDPI

Article

Chitosan Oligosaccharides Suppress Nuclear Factor-Kappa B Activation and Ameliorate Experimental Autoimmune Uveoretinitis in Mice

Sheng-Min Hsu [1], Chang-Hao Yang [2], Hsien-Yang Tsai [3], Chia-Jhen Lin [1], Yi-Hsuan Fang [4], Chi-Chang Shieh [5,*] and Shun-Hua Chen [4,*]

1 Department of Ophthalmology, National Cheng Kung University Hospital, College of Medicine, National Cheng Kung University, Tainan 704, Taiwan; shengmin@ncku.edu.tw (S.-M.H.); towawa1206@gmail.com (C.-J.L.)
2 Department of Ophthalmology, National Taiwan University Hospital, College of Medicine, National Taiwan University, Taipei 100, Taiwan; chyangoph@ntu.edu.tw
3 Department of Ophthalmology, Tzu Chi Hospital, Taichung 427, Taiwan; tc1512901@tzuchi.com.tw
4 Department of Microbiology and Immunology, College of Medicine, National Cheng Kung University, Tainan 701, Taiwan; tw7629@gmail.com (Y.-H.F.); shunhua@mail.ncku.edu.tw (S.-H.C.)
5 Institute of Clinical Medicine, College of Medicine, National Cheng Kung University, Tainan 704, Taiwan; cshieh@mail.ncku.edu.tw
* Correspondence: shunhua@mail.ncku.edu.tw (S.-H.C.) or cshieh@mail.ncku.edu.tw (C.-C.S.)

Supplementary figures

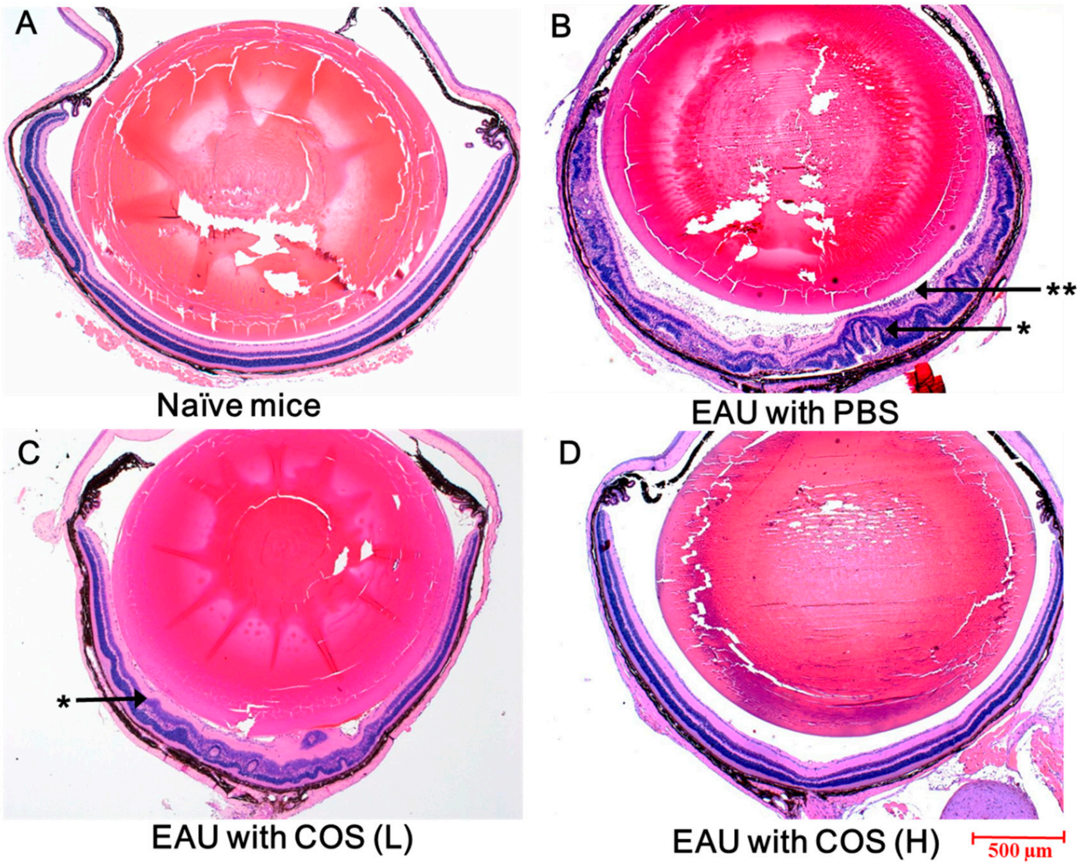

Figure S1. COS treatment reduces EAU severity in mice. Representative photomicrographs of H&E-stained slides of the vitreous cavity and whole retina of (A) naïve C57BL/6 mice without EAU induction, (B) EAU mice treated with PBS, (C) EAU mice treated with the low-dose (10 mg/kg) of COS, and (D) EAU mice treated with the high-dose (20 mg/kg) of COS. There were more than 100

infiltrative cells in the vitreous cavity of EAU mice treated with PBS (B), but less than 5 infiltrative cells in the vitreous cavity of EAU mice treated with the high-dose of COS (D). * indicates retinal folds on the retinas; ** indicates infiltrative cells in the vitreous cavity. Images are the representative results of at least four samples per group from two independent experiments.

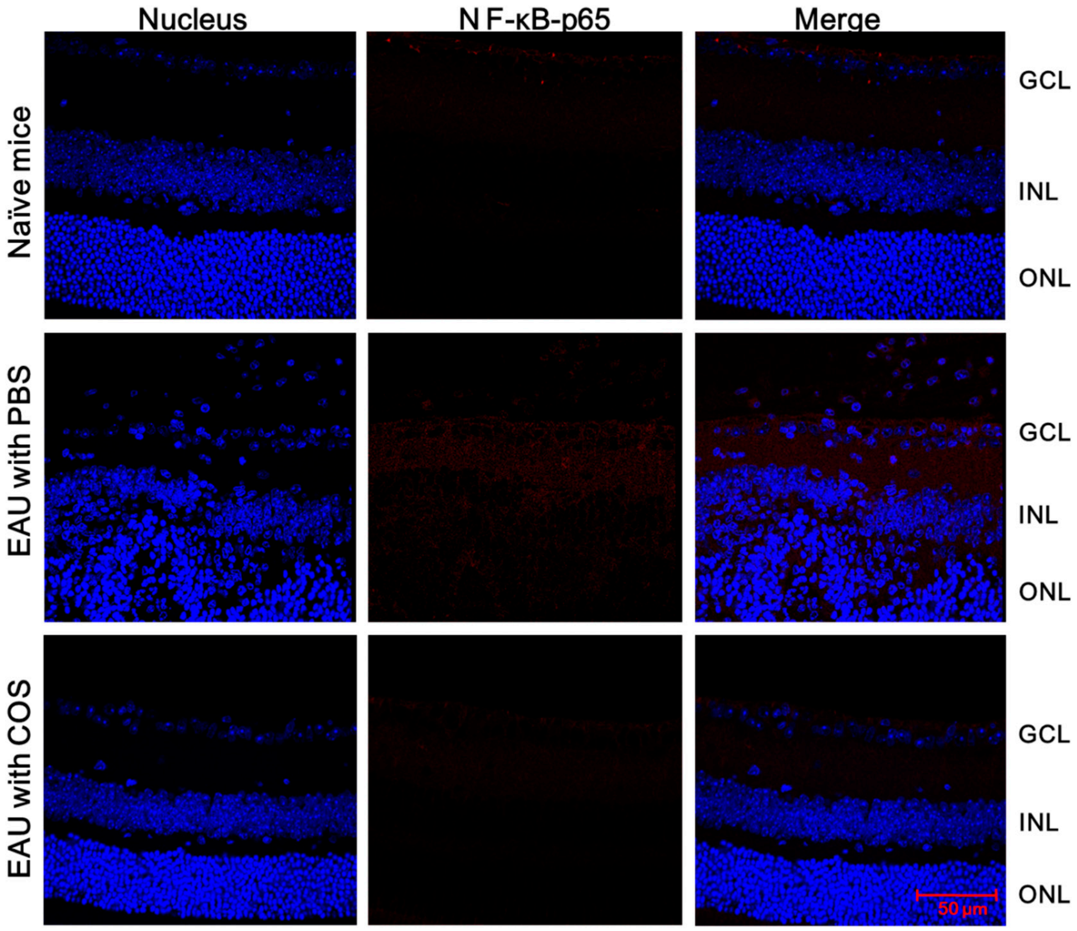

Figure S2. COS treatment reduces the nuclear translocation of NF-κB subunit, p65 in the retinas of mice with EAU induction. Eyes of mice without EAU induction (naïve) or with EAU induction were treated with PBS or COS (20 mg/kg) and monitored by immunofluorescence staining for NF-κB (p65) with antibody and for nuclei with DAPI. NF-κB (p65) was detected abundantly in the ganglion cell layer (GCL) of the retina of EAU mice treated with PBS. Images are representative of > 4 samples per group from two independent experiments. INL, inner nuclear layer and ONL, out nuclear layer.
